# CRISPR-Induced Distributed Immunity in Microbial Populations

**DOI:** 10.1371/journal.pone.0101710

**Published:** 2014-07-07

**Authors:** Lauren M. Childs, Whitney E. England, Mark J. Young, Joshua S. Weitz, Rachel J. Whitaker

**Affiliations:** 1 Center for Communicable Disease Dynamics, Department of Epidemiology, Harvard School of Public Health, Boston, Massachusetts, United States of America; 2 Department of Microbiology, University of Illinois at Urbana-Champaign, Urbana, Illinois, United States of America; 3 Thermal Biology Institute and Department of Plant Sciences and Plant Pathology, Montana State University, Montana, United States of America; 4 School of Biology and School of Physics, Georgia Institute of Technology, Atlanta, Georgia, United States of America; 5 Department of Microbiology and Institute for Genomic Biology, University of Illinois at Urbana-Champaign, Urbana, Illinois, United States of America; Boston College, United States of America

## Abstract

In bacteria and archaea, viruses are the primary infectious agents, acting as virulent, often deadly pathogens. A form of adaptive immune defense known as CRISPR-Cas enables microbial cells to acquire immunity to viral pathogens by recognizing specific sequences encoded in viral genomes. The unique biology of this system results in evolutionary dynamics of host and viral diversity that cannot be fully explained by the traditional models used to describe microbe-virus coevolutionary dynamics. Here, we show how the CRISPR-mediated adaptive immune response of hosts to invading viruses facilitates the emergence of an evolutionary mode we call distributed immunity - the coexistence of multiple, equally-fit immune alleles among individuals in a microbial population. We use an eco-evolutionary modeling framework to quantify distributed immunity and demonstrate how it emerges and fluctuates in multi-strain communities of hosts and viruses as a consequence of CRISPR-induced coevolution under conditions of low viral mutation and high relative numbers of viral protospacers. We demonstrate that distributed immunity promotes sustained diversity and stability in host communities and decreased viral population density that can lead to viral extinction. We analyze sequence diversity of experimentally coevolving populations of *Streptococcus thermophilus* and their viruses where CRISPR-Cas is active, and find the rapid emergence of distributed immunity in the host population, demonstrating the importance of this emergent phenomenon in evolving microbial communities.

## Introduction

All organisms are susceptible to infection by viral pathogens. The sheer number of viruses found in natural environments is staggering; it is estimated that 10^31^ virus particles are circulating at any time [1], [2] containing at least hundreds of thousands of genotypes [3], most of which infect bacteria and archaea. Bacteria and archaea resist infection through random mutation resulting in loss or modification of viral receptors, or through targeted defense systems such as physical blocking, restriction-modification systems, and abortive infection systems [4]–[10]. Both negative frequency-dependent selection (NFDS) and diversifying selection for microbial resistance have been suggested to result in the diversity observed in natural systems [11]–[13]. The trade-off between resistance and growth rate has become the dominant model for microbe-virus coevolution [14], with variation in fitness driving diversification of the host and resulting in the predicted coexistence of many genotypes of both hosts and viruses [15]. These theoretically predicted trade-offs have also been seen to promote diversity of both host and viral populations in experimentally evolved populations [16]–[21].

Recently the CRISPR-Cas system was experimentally shown to function as an adaptive microbial resistance mechanism, using the model organism *Streptococcus thermophilus* [22] (see reviews in [6], [23]–[32]). The CRISPR-Cas system, components of which are found in the majority of sequenced microbes [33], is comprised of short DNA fragments (spacers) flanked by palindromic repeats in repeat-spacer arrays [32]. These fragments are often identical to sequences in plasmids, viruses, and other foreign elements [34]. When a microbe containing an active CRISPR system encounters one of these foreign elements, it can add a new spacer matching a sequence in the foreign genome (protospacer) [22]. The CRISPR system can acquire spacers from many locations in a foreign genome, requiring only a short protospacer-associated motif (PAM) adjacent to the protospacer [25], [35]. Repeat-spacer arrays are transcribed, processed, and used to guide an effector complex which inactivates matched foreign genetic material on any subsequent encounter [36]. Escape mutations in protospacers prevent recognition by the CRISPR-Cas system resulting in a coevolutionary dynamic in which viruses evolve through random mutation while hosts evolve through “directed mutation” facilitated by adaptive immunity [25],[37]–[39].

We propose that crucial elements of the CRISPR system result in a diversifying coevolutionary mode that is distinct from the traditional trade-off model described above. Adaptive CRISPR acquisition of new spacers leads to the potential for competing CRISPR genotypes to emerge within a host population at the same (or similar) time – akin to the phenomena of “clonal interference” [40], [41]. The vast reservoir of protospacers in each virus creates the potential for competing host genotypes with similar (or identical) resistance phenotypes that are not necessarily subject to fitness tradeoffs between immune alleles. In contrast, viral strains are limited in potential escape mutations by fitness constraints on mutations in their genomes that can modify regulatory elements and RNA- and protein-encoding genes. In addition, each viral escape mutant only allows access to a single host immune allele potentially composing a small subset of the host population [42], [43]. We hypothesized that these differences would allow for a dramatic restructuring of the coevolutionary mode wherein many different hosts are immune to the same virus in different ways. We label this many-to-one, genotype-to-phenotype phenomenon *distributed immunity*.

We previously developed an eco-evolutionary model of CRISPR-mediated host-viral coevolution [44]. In brief, the model incorporates density-dependent Lotka-Volterra like ecological dynamics with the evolutionary introduction of new hosts and viral strains with novel genetic states. Ecological rules of interaction including host reproduction and death, viral infection of hosts and viral deactivation outside of hosts determine host and viral densities. Viral infection of hosts can lead to either host lysis or viral deactivation, which may occur with or without spacer integration. During replication, viral strains evolve through mutation generating a novel protospacer. Host immunity is determined by the presence of at least one spacer matching a virus, yet is not full-proof, i.e., there is a small chance that a host with a matching spacer to an infecting virus will not be immune [44]. In simulations of our model, host and viral populations oscillate in abundance over short time scales, whereas host and viral genotype composition changes over long time scales, mediated by coevolutionary adaptation. A comparison of this and other models of CRISPR-mediated coevolutionary dynamics (e.g., [45]–[49]), whose exact dynamics depend on the specific molecular, ecological and evolutionary parameters can be found elsewhere [44], [50].

Within our model, examining the diversity of the host population at each maximum in total host population abundance (host peaks), we observed two types of emergent population dynamics: (i) near selective sweeps by novel or recurring strains and (ii) simultaneous growth of phenotypically similar but genotypically diverse groups of strains which we termed coalitions [44]. Although the diversification of host populations with CRISPR immunity had been noted previously [44], [45], [48], [49], [51], in this paper, we present a metric, *population-wide distributed immunity* (PDI) to quantify distributed immunity in a population, to examine how distributed immunity varies over time and to determine how this evolutionary mode affects the coevolutionary dynamic. We used simulated data from our model to: (i) determine when coalitions are characterized by distributed immunity; (ii) identify conditions under which distributed immunity is the dominant evolutionary mode in a simulation; and (iii) quantify the effects of distributed immunity on host-viral relationships by examining diversity and stability of host and viral populations. Finally we determined that the diversity exhibited in an experimental host-viral community is associated with distributed immunity.

## Results

### Quantifying distributed immunity

**Figure 1:**
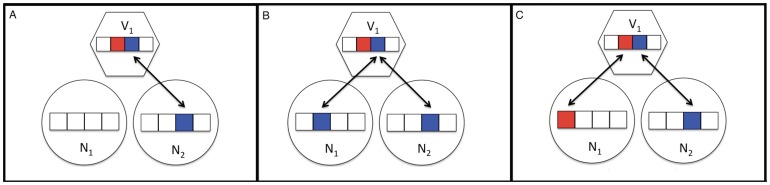
Population distributed immunity (PDI) depends on immunity relationships between hosts (circles) and viruses (hexagons). Immune elements are denoted as linear arrays of boxes. PDI is the sum of contributions (δPDI) calculated amongst triplets of two hosts and one virus, adjusted by their population proportions, as follows: (A) δPDI = 0 when only one (or neither) hosts in a triplet match the virus as *R(N_1_,V_1_) = M(N_1_,V_1_) = 0*, *R(N_1_,V_1_) = M(N_1_,V_1_) = 1*,and *R(N_1_,N_2_,V_1_) = 0*. (B) δPDI = 0 when both hosts match the virus with the same spacer as *R(N_1_,V_1_) = M(N_1_,V_1_) = 1*, *R(N_1_,V_1_) = M(N_1_,V_1_) = 1*,and *R(N_1_,N_2_,V_1_) = 0*. (C) δPDI = N_1_N_2_V_1_{1-[|N_1_-N_2_|/max(N_1_,N_2_)]} when both hosts match the virus via different spacers ass *R(N_1_,V_1_) = M(N_1_,V_1_) = 1*, *R(N_1_,V_1_) = M(N_1_,V_1_) = 1*,and *R(N_1_,N_2_,V_1_) = 1*. Identical colors, indicated by arrows, represent matching spacer-protospacer pairs. White protospacers and spacers are unique.

*Distributed immunity* denotes the emergent phenomenon in which multiple immune alleles coexist within and between hosts. When these alleles are distributed between different hosts that have CRISPR-Cas resistance, then multiple hosts have similar immune phenotypes yet have distinct, coexisting associated CRISPR genotypes. To measure the impact of distributed immunity, on each population, we developed a metric called population-wide distributed immunity (PDI) in which CRISPR-Cas immune relationships of all host-host-viral strain triplets are tested to determine if the two host strains contain spacers matching different protospacers on the same viral strain (Figure 1, see Methods for details of the calculation). The intuition behind our metric is that all triplets contribute positively to PDI when both hosts are immune to the virus by means of distinct spacers matching the virus. In the case where both hosts are immune to the virus but via the identical spacer, the immunity is not distributed throughout the population and thus does not contribute to PDI. Although phenotypically immunity via identical or distinct spacers is equivalent, the varied genotypes may follow different evolutionary pathways. For example, when PDI is high, mutation of a single protospacer does not permit escape in the majority of the host population. However, when PDI is low, a single protospacer mutation may lead to viral escape in most of the host population. The degree of contribution by each triplet depends on the product of the relative abundance of the host strains and viral strain and immunity between the host and viral strains (see Methods for details of the calculation). The maximum PDI for a population at any time increases with the number of host strains (with n host strains the maximum is 1-1/n) and is only obtainable when the following hold: there are at least two alleles that confer immunity to the viral strains, all host strains are immune to viral strains, and the abundance of each host CRISPR allele is equal (Figure S1). Note that the abundance of the viral strains does not affect the potential for PDI (see SI text for further discussion).

**Figure 2:**
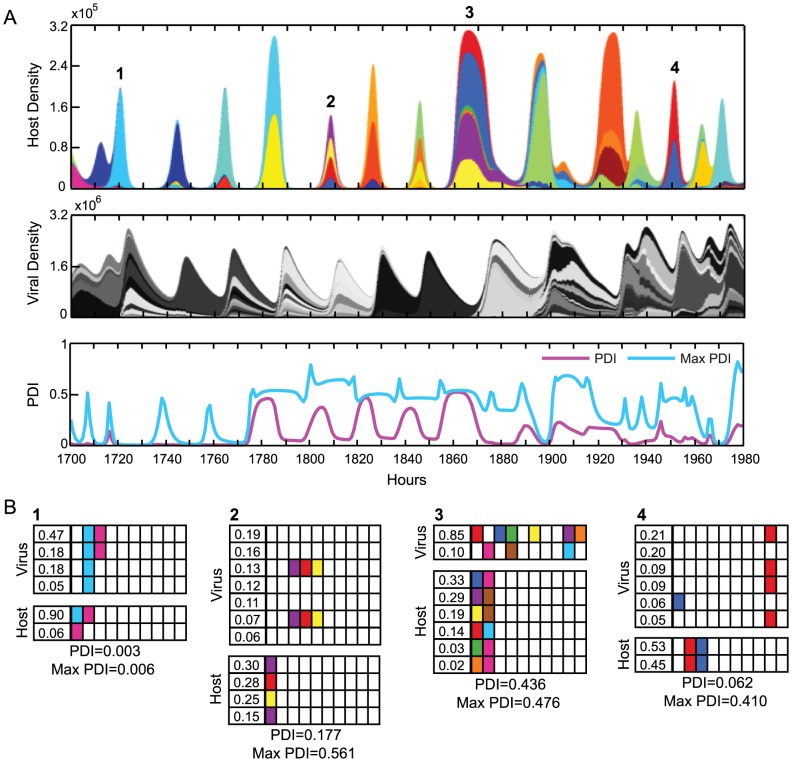
Host populations exhibit periods of different coevolutionary dynamics. (A) Population dynamics of the host (top) and virus (middle) and PDI (bottom) from a representative simulation. Each color represents a host or viral strain with a unique spacer or protospacer set and is proportional in height to the strain proportion in the population; colors repeat when not touching. (B) Spacer-protospacer matches between major host and viral strains at four time points as examples of single-strain dominance (**1**), coalitions with low immunity (**2**), coalitions with high PDI (**3**), and coalitions with high immunity but low PDI (**4**). The spacer and protospacer composition of each host or viral strain, respectively, is listed horizontally. The number in the first column indicates the proportion of each strain in the population, while the remaining boxes represent the spacer or protospacer state. Host strains making up less than 2% and viral strains making up less than 5% of the population, which only have minor impact on the calculated PDI, are omitted for space. Matching colors in host and viral boxes indicate a spacer-protospacer match. White boxes are spacers or protospacers without a match. Model parameters are standard parameters in Table S2.

**Figure 3:**
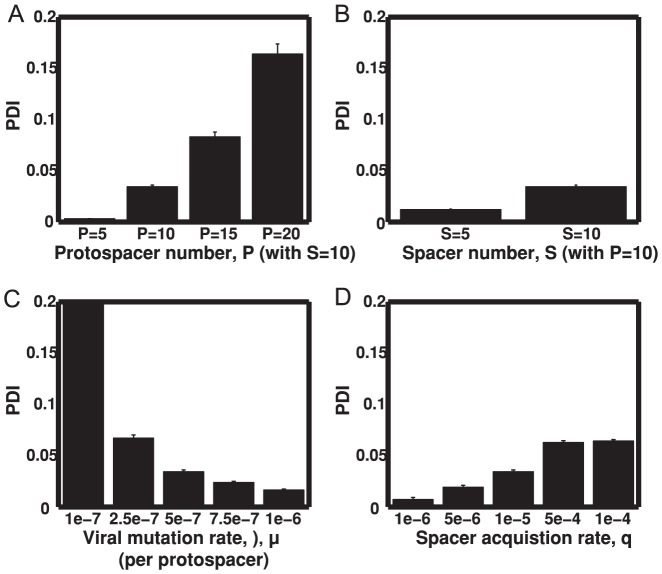
PDI is elevated at high protospacer number and low viral mutation rate. Measured PDI in numerical simulations is shown with varying (A) protospacer number; (B) host acquisition rate; (C) viral mutation rate; (D) spacer number. Bars (and lines) represent mean (and SEM) of PDI of replicate simulations, with each replicate represented by the median value across the final 500 hours of that single simulation. Unless varied, parameters are S = 10, P = 10, q = 10^−5^, μ = 5×10^−7^. Using analysis of variation for unbalanced data all pairwise comparisons of mean PDI are significant at p<0.001 except comparisons between: μ = 5×10^−7^ and μ = 7.5×10^−7^ (p<0.01) in (C); μ = 7.5×10^−7^ and μ = 10^−6^ (not significant) in (C); and q = 5×10^−5^ and q = 10^−4^ (not significant) in (D).

**Figure 4:**
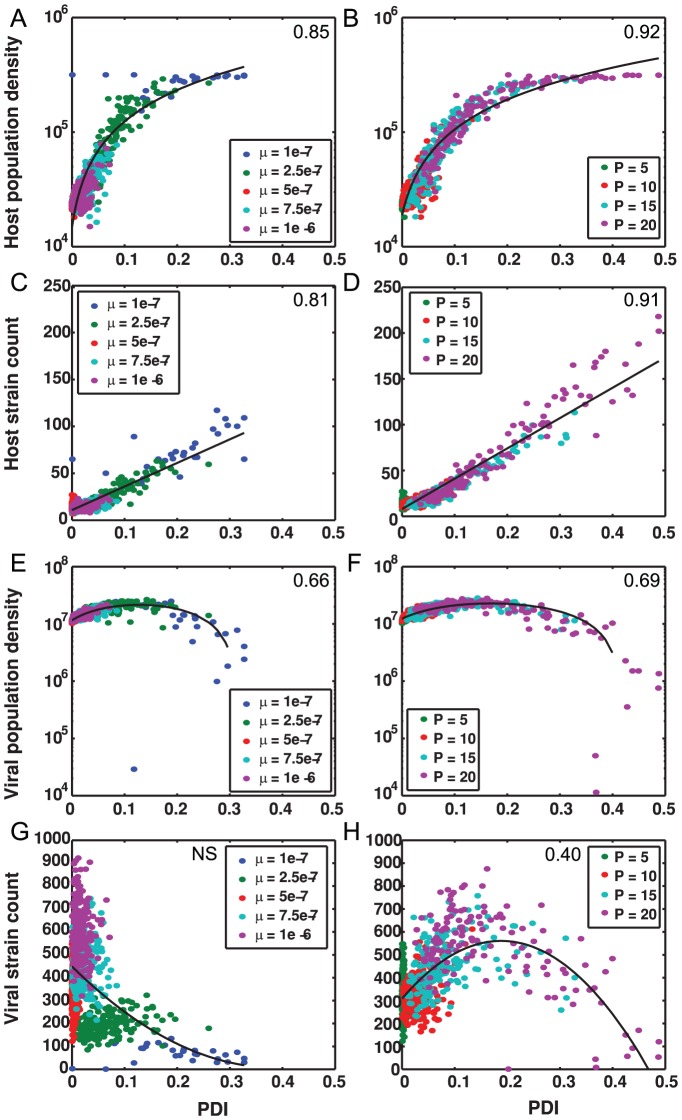
PDI and population measures when varying mutation rate and protospacer number. High PDI is associated with (A–B) increases in host population density, (C–D) increases in host strain count, non-monotonic changes (E–F) in viral population density, and non-monotonic changes (G–H) in viral strain count. Left column is varying mutation rate, μ, and right column is varying protospacer number, P. Unless varied, parameters are S = 10, P = 10, q = 10^−5^, μ = 5×10^−7^. Each point is the median from the last 500 hours of a single simulation. Note that both viral population density and viral strain count are unimodally related to PDI, with the lowest levels of both viral population density and strain count occurring at high PDI. The Spearman rank correlation coefficients of all comparisons, noted in the upper right corner of Figure panels, are significant at p<0.001. These relationships, including those that are non-monotonic, are discussed further in the main text.

In the simulated eco-evolutionary dynamics of hosts and viruses [44], we find that PDI varies through time (Figure 2). PDI is typically highest just prior to peaks in host population density and drops to at or near zero in between (Figure 2). Every peak of host density does not contain high PDI, even if its potential maximum PDI is high, and in our simulations we find that measured PDI is well below the potential maximum. Low PDI results from (i) unevenness of the host population (Figure 2b-1, Figure S1), (ii) a large fraction of the hosts lacking immunity to the viral population (Figure 2b-2) or (iii) the majority of hosts having immunity to the viral population via the same spacer (Figure 2b-4). In contrast, high PDI occurs when multiple hosts have unique spacers to the same viral strains. This can occur when a dominant host strain diversifies via the acquisition of unique spacers to the same viral strain (Figure 2b-3). Across all simulations, the PDI at host peaks ranges from 0 to 0.7203 with an overall mean of 0.0710. We find no direct, predictable relationship between the abundance of host and viral populations at their peaks in relation to the concurrent value of PDI within a single simulation. In contrast, we hypothesize that PDI functions to alter the future host and viral dynamics within a community. Diversified hosts (with a high PDI) may affect the composition and total density of virus populations that recur in the next peak in host density or much later. This is due to the complexity and diversity of both host and viral populations in which a particular diversified host can be targeted by divergent low abundance viruses that were created much earlier.

### Parameters that increase population-wide distributed immunity

To determine how biological parameters might influence the evolutionary mode across a simulation toward or away from distributed immunity, we altered four parameters that vary between microbial and viral strains: viral mutation rate, *μ*; spacer acquisition rate, *q*; maximum host spacer number, *S*; and viral protospacer number, *P*. To avoid the period of transient dynamics occurring at the initiation of the simulations from a single viral and single host strain, we measure median PDI in the last 500 hours of each simulation, where the host spacer locus is filled and both host and viral diversity are most regular (see File S1, Figure S2). Comparing the population dynamics between sets of simulations with varying parameters, we found that average PDI across the simulations increases when viral mutation rate decreases and when the number of relative protospacers increases (Figure 3). There are also increases in PDI when the spacer acquisition rate increases and the number of spacers increases, but PDI above 0.1 is rarely seen (Figure 3). The highest average PDI is seen with high relative protospacer number (P = 20) and low viral mutation rate (μ = 10^−7^) while lowest average PDI occurs with low relative protospacer number (P = 5) and low spacer acquisition rate (q = 10^−6^). Increases in average PDI result from coevolutionary dynamics that include more host population peaks with higher PDI, rather than from an increase in PDI when host populations are not near their peak values.

### Population-wide distributed immunity is associated with individual distributed immunity

In simulations with a higher average PDI, we observed an additional dynamic where individual host genotypes contain multiple spacers matching the same viral strain at distinct protospacers. This represents an analogous form of distributed immunity, albeit within a single host. Since this will have similar evolutionary effects as PDI, we quantify the average per host immunity to viral strains with a new metric denoted as individual distributed immunity (IDI). IDI is equal to the average number of distinct matching spacers between each pair of viral and host strains (see Methods for details of the calculation). When IDI is greater than one, the host population is on average immune in multiple ways to the viral population due to targeting multiple regions of the viral genome. We find that there is strong correlation between PDI and IDI (Figure S3) and, as with PDI, there is high IDI with low viral mutation rate and high protospacer number (Figure S4). Hereafter, we collectively refer to PDI and IDI as DI.

### Elevated distributed immunity is associated with increases in host diversity, density, and stability

**Figure 5:**
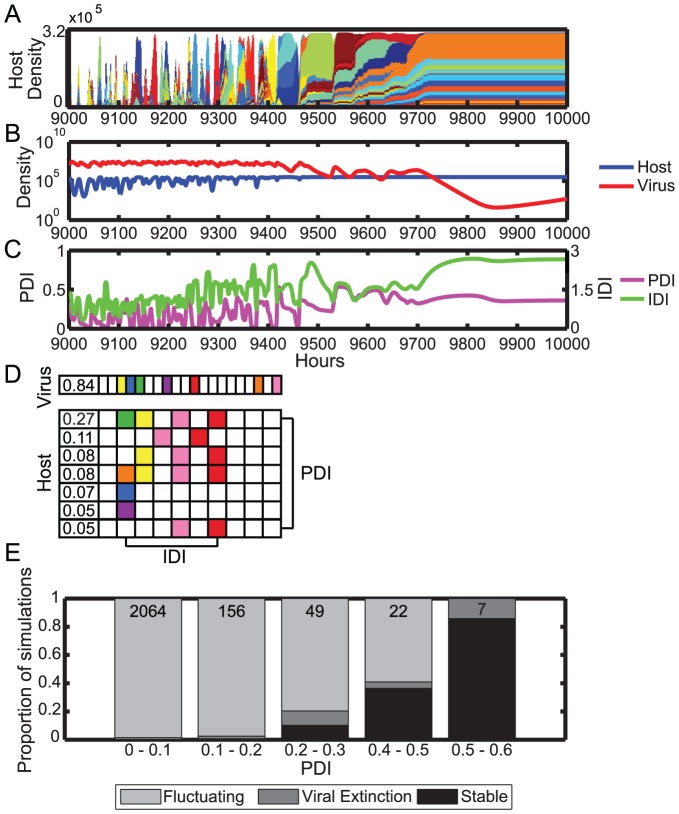
Host stability in a high DI population. (A) Plot of host dynamics for a representative model simulation containing an extended period of host population stability. Each color represents a host strain with a unique spacer set and color height is equal to population proportion of the strain; colors repeat when not touching. (B) Total population density in log-scale of host (blue) and virus (red) strains. (C) PDI (magenta, left y-axis) and IDI (green, right y-axis) metrics. (D) Spacer-protospacer matches at 9800 hours. The spacer and protospacer composition of each host or viral strain, respectively, is listed horizontally. The number in the first column indicates the proportion of each strain in the population, while the remaining boxes represent the spacer or protospacer state. Strains making up less than 5% of the population are omitted for clarity. (E) Numbers at the top of each bar designate the total number of simulations in each bin. A simulation is denoted as “stable” when the host population remains above 3e5 (close to carrying capacity) for at least 100 consecutive hours, and as “viral extinction” if the simulation ends prior to the designated endpoint due to reaching a viral population size below our density cutoff of 0.1/mL. Comparisons of subsampled data for stable and viral extinction show significant differences between means of all PDI bins (except between 0–0.1 and 0.1–0.2 stable simulations) and all IDI bins (except between1.8–2.4 and 2.4–3.0 stable simulations).

Having identified conditions under which simulations with high levels of distributed immunity are linked to changes in host-virus relationships, we investigated possible consequences of these altered interactions. We found that simulations resulting in high levels of distributed immunity are correlated with increased host strain count and population density (Figure 4A–D). We find a much stronger association between DI and these population level indicators than when evaluating the statistical relationship between mutation rate and protospacer number alone. For example, the Spearman rank correlation coefficient between host population density and PDI is 0.84 whereas it is −0.31 and 0.49, when evaluated against mutation rate and P, respectively (all p<0.001). Similarly, the Spearman rank correlation coefficient between host strain count and PDI is 0.78 whereas it is −0.26 and 0.27, when evaluated against mutation rate and P, respectively (all p<0.001). The data collapse of host population density and host strain count as a function of PDI from simulations with different governing parameters is apparent in Figure 4A–D. Investigating simulations where distributed immunity has a strong effect (high DI), we also observed extended periods of high density, stable host populations (see time points between 9700–10000 in Figure 5A–C for a typical example). Periods of stable host-controlled dynamics occur exclusively in parameter sets which have higher DI: P = 15, P = 20, and μ = 10^−7^, and the proportion of simulations which exhibit extended stable periods increases with increasing DI (Figure 5E, black bars). The finding of extended stability is not driven solely by the extended high host density; this pattern is observed whether DI is measured at all time points (as in Figure 5E), or only at host density peaks.

### Elevated PDI is associated with decreased viral diversity and density

**Figure 6:**
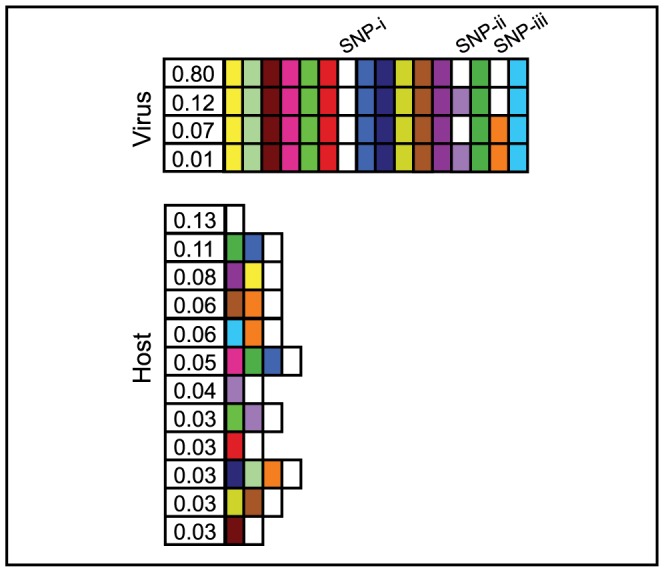
PDI and IDI estimated in a population of *Streptococcus thermophilus* and its phage 2972. Data from [38] (accession number SRA049615). Virus protospacer and host spacer states are shown after one week of experimental coevolution. Matching colors in host and viral boxes indicate a spacer-protospacer match. White boxes are spacers or protospacers without a match. All viruses are shown; host strains that make up less than 3% of the population are not shown for clarity. Protospacer positions for which there is no match between any virus and the hosts shown are omitted.

In contrast to the increases in host population density and host strain count as PDI increases, the trends for viral population density and viral strain count are non-monotonic (Figure 4E–H and Figure S5). At lower PDI (PDI<0.2) increases in PDI correlate with increases in viral population density and weakly correlate with increases in viral strain count (Figure 4 and Figure S5). The observed viral population increases are also correlated with increases in host population size and host immunity (Figure S6). Although immunity is increasing, it is still relatively low, suggesting that individual viral strains can continue to grow on subsets of the total host population. Simultaneously, as PDI increases, the host population is also increasing, so that each subset of hosts that viruses can infect is actually larger than at lower PDI. At higher PDI (PDI>0.2), increases in PDI correlate with decreases in viral population density and viral strain count (Figure 4). Beyond PDI = 0.2, increases in host population size and immunity no longer correspond to higher viral densities. This decrease in viral density is consistent with the fact that the proportion of hosts that viruses can infect (HVI, see Methods for details of the calculation) decreases as DI increases, and HVI is significantly lower in simulations with higher DI (Figure S7). Accompanying decreases in viral population sizes we find that the proportion of simulations in which viruses go extinct increases with increasing DI (Table S1 and Figure 6, dark gray bars). Parameter sets with the highest DI, P = 20 and μ = 10^−7^, result in viral extinction in 10% and 12% of simulations with filled loci, respectively, the highest rates of extinction of any parameter set (Table S1). Considering simulations in which the CRISPR locus does not fill before the last 500 hours, 90.7% end in viral extinction, including 94.3% and 91.6% of P = 20 and μ = 10^−7^ simulations, respectively. Nearly all simulations with lower DI reach a full spacer locus prior to the final 500 hours (Table S1).

### Elevated distributed immunity identified in an experimental viral-host community

We examined whether the dynamic of distributed immunity observed in simulations is consistent with patterns observed in experimental microbial communities in which both virus and host sequence is known. To do so, we estimated DI within an experimental set of host and viral populations. A quantitative assessment of the contribution of the relative DI to the maintenance of diversity in natural microbial populations is not possible in most studies, as the contemporary virus population is not typically sequenced. Despite technical challenges to date in testing distributed immunity in natural populations, studies in laboratory populations offer an opportunity to measure distributed immunity. Numerous studies in laboratory populations have shown that upon challenge by a single phage, multiple *S. thermophilus* genotypes emerge with different spacers providing immunity [22], [25], [26], [37], [38], [52]. For our analysis, we used data from Sun *et al.* [38], the only study with both sequences and abundances from the entire coevolving host and viral populations as required to measure DI. In this study, a laboratory-coevolved population of *Streptococcus thermophilus* and its phage 2972 was found to exhibit rapid spacer addition as well as phage CRISPR escape mutations. After 1 week of co-culture, the host had added 43 new spacers to one CRISPR locus, and three viral mutations in targeted protospacers or PAMs were detected [38]. Given the diversity of new spacers matching a small pool of viral types, we estimated a high value of PDI for these populations. Using populations reconstructed from spacer-containing reads and viral SNP distributions (Figure 6, see Methods), the value of PDI after 1 week of coevolution was 0.4331, out of a maximum possible PDI of 0.5933. This estimate of elevated PDI complements Sun *et al*.'s [38] observation of multiple acquisitions of distinct CRISPR escape mutants, and suggests a population-level effect that may act synergistically with individual host-viral interactions. Note that this PDI value is larger than the median PDI in 99.8% and the highest observed PDI in 75.9% of all simulations we conducted. The value of IDI, 1.2264, was higher than the median IDI in 97.7% and the highest observed IDI in 58.7% of simulations.

## Discussion

We have explored the immune dynamics resulting from a computational eco-evolutionary model driven by CRISPR-mediated immunity. The model demonstrates how a host-viral community can evolve a complex structure where different hosts are immune to the same virus as a result of immunity conferred by different immune alleles, which we have quantified as distributed immunity. Immunity relationships between hosts and viruses with distributed immunity may appear similar from the phenotype level to relationships lacking distributed immunity; however, the underlying genetic diversity present in distributed immunity changes the dynamics of coevolution. In particular, during periods of elevated distributed immunity, the host population is diverse and stable while the viral population is restricted in the number and extent of possible beneficial mutations and is prone to extinction. The stable maintenance of multiple non-dominant genotypes that accompanies distributed immunity is likely facilitated by NFDS. The generation of distributed immunity and the selective mechanisms of NFDS may work together to promote diversity.

Several CRISPR models have previously observed diversity in host spacer content both at an individual and population level [44], [45], [48], [49], but understanding that diversity has been a recent exploration. Although Iranzo *et al.* [49] established several population-level findings, such as CRISPR immunity promoting the coexistence of viruses and hosts at intermediate viral mutation rate and the lack of increased viral diversity with CRISPR immunity, they did not attempt to expound upon these findings, which they labeled counterintuitive. Our model, even with its reduced complexity as we ignore populations lacking CRISPRs, is able to reproduce these results and offer an explanation for them via distributed immunity. Here, we have demonstrated that the consequences of viral protospacer number and mutation rate as well as host spacer acquisition rate and spacer number on the population dynamics can be explained as acting through distributed immunity thereby linking the molecular and evolutionary mechanisms to the eco-evolutionary dynamics that have been observed. Since distributed immunity only requires some of the spacers to be distinct, it is consistent with a previously posed model where random deletion lead to selective sweeps of trailer-end spacers [48].

CRISPR-Cas diversity varies greatly among systems. At one end of the spectrum are the slowly-evolving CRISPR-Cas systems of *Escherichia* and *Salmonella*, where estimates indicate that strains that have diverged in the last thousand years have identical CRISPR loci [53]. At the other end are natural populations exhibiting high CRISPR-Cas diversity, including the human gut microbiome [54], *Yersinia pestis* plague foci [55], and hot spring populations of *Sulfolobus islandicus* [56], [57]. Notably, in the case of *S. islandicus*, these archaeal populations do not contain a dominant genotype or display evidence of selective sweeps over a ten-year interval [57] but maintain diversity at both the leader and trailer ends of the CRISPR loci over time. Some natural populations demonstrate evidence of past selective sweeps in the form of conserved trailer-end spacers, particularly populations of acidophilic microbes found in acid mine drainage [48], [58], [59]. The difference between the immune structures of different microbial populations may be driven by differences in the extent of distributed immunity within populations, differences in the levels of reassortment of CRISPR alleles between strains in different populations [57], or the action of other host defense systems operating along with CRISPR-Cas immunity.

Indeed, our model suggests that the biology of CRISPR-Cas system might define the resulting level of diversity observed in natural populations. We show that the number of protospacers, viral mutation rate, and host acquisition rate all significantly influence the level of distributed immunity in a way that would result in different immune structures in natural populations. These factors have been shown to vary in natural microbial populations. For example, in microbes with active CRISPR-Cas defense, the number of protospacers is determined by both the length of the viral genome and the length and sequence of the PAM sequences, which direct acquisition and interference. We infer that protospacer number is positively correlated with distributed immunity because at higher protospacer numbers it is easier for hosts to acquire multiple spacers to the same virus (higher IDI) and for different hosts to acquire different spacers (higher PDI). We hypothesize that microbial hosts utilizing shorter PAMs or that are infected by viruses with larger genomes are more likely to display a diversified immune structure that is consistent with distributed immunity. Variation in viral mutations rates has also been observed in natural populations. For example, it has been suggested that thermophiles and their viruses have lower mutation rates than their mesophilic counterparts [60], [61]. Our model suggests that this is consistent with data showing that the thermophilic archaeon *S. islandicus* appears to maintain a stable diversified population over time [56], [57]; however, this hypothesis must be explicitly tested. Finally, in this study we did not explore variation in the probability that CRISPR immunity fails such that a host cell does not recognize and clear a virus for which it has a matching spacer. Such failure may result in the proliferation of a virus to which there exists some immunity in the population. Given our previous analysis showing the relatively minor effects of such failure on resulting dynamics [44], we do not expect significant effects of the stochastic failure of host spacers on distributed immunity, at least in the range of failure values observed experimentally [22]. However, in the case of exposure to plasmids rather than viruses, such failure may permit the exchange of genetic material between hosts [62]. Under conditions when genetic exchange is advantageous (e.g., in the presence of many beneficial plasmids [63]) then the occurrence of distributed immunity may result, even if seemingly unfavorable, to protect against virulent viruses.

Although natural population data is not yet available to employ our novel metrics PDI and IDI for quantifying distributed immunity, we have quantified this evolutionary mode in an experimental population. Qualitatively, Sun *et al.* [38] observed rapid transition from clonal to diversified in both host and viral populations as a result of CRISPR-Cas immunity. We demonstrated that this diversification also exhibited rapid emergence of DI and hypothesize that our finding of highly elevated PDI in Sun *et al.* [38] may be due, in part, to the relatively large number of protospacers in the genomes of phage (associated with replete PAMs), as compared to the use of low number of protospacers (P = 5–20) in our models due to computational constraints. This hypothesis is further supported by our simulation results where DI increases as we increase protospacer number (see Figure 3A). We predict that in this system when the *S. thermophilus* hosts exhibit distributed immunity, viral populations will be smaller, less diverse and more prone to extinction. We consider it an important future goal to extend the DI analysis of *S. thermophilus* and phage to systems in which host and viral metagenomes are available to further quantify the variation of DI in natural populations.

A better understanding of CRISPR-mediated coevolutionary dynamics will have important implications for medical applications for example those seeking to target microbial pathogens with phage therapy. In addition, our model suggests possible optimal strategies for engineering stable microbial communities immune to phage attack such as those used in biofuels production or other industrial applications. Finally, CRISPR immunity serves as an interesting model system in which to study the broader effects of diversified immunity on pathogen evolution. Such diversity impacts the trajectory of host-virus coevolution in microbes mediated by CRISPR-Cas immunity. Further understanding how distributed immunity affects the evolutionary path of populations may yield insight into the effects of host immune diversity in microbial communities and other systems.

## Methods

### Model information and statistical analyses

We use the model introduced in Childs *et al.* [44] to generate our simulation data. Briefly, in the model, ecological host-viral dynamics are combined with the introduction of new host and viral strains through changes in the CRISPR space and protospacer states. Hosts may acquire new spacers during viral infection, and viruses may mutate to novel protospacers during replication. Host immunity towards an infecting virus requires the presence of at least one spacer matching a viral protospacer, but is not full proof. The population dynamics of host and viral strains are deterministic but the incorporation of hosts' spacers and mutation of viral protospacers occurs stochastically. Further details of the model are reviewed in the supplemental information with the parameters used in Table S2. Although this paper focuses on four parameters (protospacer number, spacer number, viral mutation rate, spacer acquisition rate), Childs *et al.* [44] more thoroughly tests dependencies of model dynamics on other parameters. Due to the stochastic nature of our model, the parameter regions surveyed were limited by computational cost. All results presented are averages of 200 replicate simulations, unless otherwise noted (Table S1), with each replicate represented by the median value across the final 500 hours of that simulation. One hour is equivalent to the inverse of the growth rate – what we denote here as a typical host generation time. Simulations were excluded from population averages whenever the spacer states did not contain the maximum number of spacers (full locus) throughout the final 500 hours of simulation or whenever the viral population fell below our density cutoff before the locus was filled (Table S1).

For each of the four parameters varied (protospacer number, spacer number, viral mutation rate, spacer acquisition rate), measurements from replicates at each parameter value tested were grouped. The means of replicate PDI and IDI measurements were compared using analysis of variation for unbalanced data (data from Figure 3 and Figure S4).

The Spearman rank correlation coefficients were determined for variations in each parameter between PDI, host population density, viral population density, host strain count, viral strain count, and IDI (data from Figure 3, Figure S3 and Figure S5). The Spearman rank correlation coefficients were also determined for variations in PDI, host population density and immunity combining all parameter sets (data from Figure S6). R^2^ values were determined for correlations between HVI and PDI, and between HVI and IDI (data from Figure S7).

The data collapse of host and viral output variables, as a function of PDI, from simulations with different governing parameters is apparent in Figures 4 and Figure S5. To test for correlations, linear R^2^ values were determined for variations in each parameter between PDI, host population density, viral population density, host strain count, viral strain count and IDI for variations in each parameter (data from Figure 4, Figure S5). Despite significant linear correlation in almost all cases, except between PDI, host strain count and viral strain count when varying S, it was evident upon inspection that the relationships between PDI and viral population density and viral strain count were better described by non-linear functions, particularly quadratic functions. To quantify this, we fit a quadratic model for viral output parameters and compared the quality of fit to a linear model using AIC; the relationship of all PDI and viral output statistics were better fits as demonstrated by lower AIC values except for PDI and viral strain count when varying S where both linear and quadratic fits were not significant (See Table S3).

To compare the proportion of simulations that are stable, fluctuating, or end in viral extinction, 10,000 random subsamples of 230 simulations (10% of the total simulations with filled loci) were taken. The mean proportions of simulations in each bin that fell into the stable or viral extinction category were compared using analysis of variation (data from Figure 6). We define a population to be stable when the host population exceeds 3e5 for more than 100 hours (approximately 95% of the carrying capacity).

### Population-wide distributed immunity (PDI)

To quantify the population-level distribution of immune alleles between hosts with similar immune phenotypes but distinct CRISPR genotypes, we compare all triplets of two host strains and a viral strain. We determine which triplets contain distinct spacers matching protospacers in the virus to quantify PDI as follows:
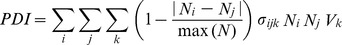


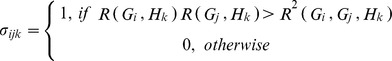
where *N_i_* is the population proportion of the *i^th^* host strain, *V_k_* is the population proportion of the *k^th^* viral strain, *G_i_* is the set of spacers belonging to the *i^th^* host strain, *H_k_* is the set of protospacers belonging to the *k^th^* viral strain, *R(G_i_,H_k_)* determines the number of matching spacers and protospacers between the states *G_i_* and *H_k_*, and *R(G_i_,G_j_,H_k_)* determines the number of matching spacers and protospacers between all the states *G_i_*, *G_j_* and *H_k_*. Further, max(*N*) denotes the maximum proportion of any given host strain in the population.

Triplets with matching spacers and protospacers contribute to PDI via the function *σ*. The relative of abundance of the strains from a triplet determines the level of contribution of that triplet to PDI. The total value of PDI is weighted by host strains at or similar to the size of the dominant host strain in order to minimize the summed contribution of numerous strains found at low proportion.

### Individual distributed immunity (IDI)

We introduce individual distribution immunity to quantify the distribution of immunity within hosts, in contrast to PDI, which quantifies the distribution of immunity between hosts. IDI is the average number of spacers per host matching the viral population:
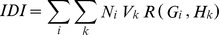
where the host proportion (*N_i_*), the viral proportion (*V_k_*), the host spacer state (*G_i_*), the viral spacer state (*H_k_*), and the number of matches between spacer and protospacer states *R(G_i_,H_k_)* are defined as in PDI.

### Hosts that Viruses can Infect (HVI)

The average proportion of hosts that viruses can infect is quantified by HVI:
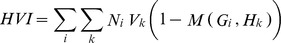
where *M(G_i_,H_k_)* determines the presence or absence of matching spacers and protospacers between the states *G_i_* and *H_k_*. The host proportion (*N_i_*), the viral proportion (*V_k_*), the host spacer state (*G_i_*), the viral spacer state (*H_k_*) are defined as in PDI.

### Experimental population DI calculations

Sequencing reads from the Sun *et al.* study [38] (accession number SRA049615) containing at least two novel spacers, or at least one novel spacer plus ancestral spacers or leader sequence were considered. Reads were grouped by spacer content; where trailer-end sequence information was not available, the locus was assumed to have the same trailer-end spacers as other reads with similar leader-end spacer content (Figure S8). If trailer end spacers could not be inferred in this way, the trailer end was assumed to contain only spacers fixed in the population (Figure S8). Each unique set of spacers was considered a host strain; the proportion of reads matching each strain was used for the proportion of each strain in the population (*N_i_* and *N_j_*) for calculation of PDI and IDI. Assuming similar CRISPR loci whenever possible maximizes the number of reads grouped into each CRISPR-type and prevents overestimation of PDI.

Frequencies of three phage mutations in protospacers or PAMs identified by Sun *et al.* [38] were confirmed using breseq [64](available online at http://barricklab.org/breseq). Each possible combination of SNPs was considered a different viral strain. To determine the proportion of phages with each combination of SNPs (SNP-i only, SNP-i and SNP-ii, SNP-i and SNP-iii, or all three SNPs), each mutation was considered an independent event and the probability of each combination was calculated. These proportions were used for *V_k_* in the PDI and IDI equations. Otherwise, PDI and IDI were calculated as in simulated populations.

## Supporting Information

Figure S1 **Maximum possible PDI changes with the number of host strains.** The maximum attainable PDI is determined by the number of host strains, the evenness of the host abundances and requires all host strains are immune to all viral strains. Maximum PDI increases towards one when all hosts have equal abundance (blue). When one host dominates, for example 50% of the population (green) or 90% of the population (red), and all other hosts have equal abundance, the maximum PDI is significantly reduced.
(EPS)

Figure S2 **Early time course of a representative simulation with standard parameters listed in Table S2.** Despite seeding with a single host and viral strain, many strains rapidly appear as result of the ever-changing immunity structure. Thick lines at the top of panels A and B are total population density; thin lines are population density of individual host strains (blue lines, A) and viral strains (red lines, B). During the initial hours there is more defined population strain structure when the average spacers per host is low (C).
(EPS)

Figure S3 **PDI is positively correlated with IDI.** Each point is the median from last 500 hours of a single simulation varying (A) protospacer number, P; (B) spacer number, S; (C) viral mutation rate, μ; (D) host spacer acquisition rate, q. Unless varied, S = 10, P = 10, q = 10^−5^, μ = 5×10^−7^. R^2^ correlation coefficients, noted in the upper-right corner of figure panels, of all comparisons are significant at p<0.001. Correlations are depicted with solid black lines.
(EPS)

Figure S4 **IDI varies with: (A) protospacer number; (B) spacer number; (C) viral mutation rate; (D) spacer acquisition rate.** Unless varied, S = 10, P = 10, q = 10^−5^, μ = 5×10^−7^. Bars (and lines) are mean (and SEM) of IDI of replicate simulations, with each replicate represented by the median value across the last 500 hours. Using analysis of variation for unbalanced data all pairwise comparisons of mean PDI are significant at p<0.001 except in (D) where all pairwise comparison with q>1e-6 (not significant).
(EPS)

Figure S5 **PDI and population measures when spacer acquisition rate and spacer number are varied.** PDI is only weakly correlated, if at all, with host population density (A–B), host strain count (C–D), viral population density (E–F) and viral strain count (G–H) across variation in spacer acquisition rate, q (left column), and spacer number, S (right column). Unless varied, S = 10, P = 10, q = 10^−5^, μ = 5×10^−7^. Each point represents the median of the last 500 hours in a single simulation. Linear R^2^ correlation coefficients (A–D) and quadratic R^2^ correlation coefficients (E–H), noted in the figure panels, of all comparisons are significant at p<0.001 except PDI with host strain count (in D) and PDI with viral strain count (in G) when spacer acquisition rate is varied. Correlations are depicted with solid black lines (A–D) and curves (E–H).
(EPS)

Figure S6 **Low PDI (<0.2) is correlated with increases in immunity (A) and host population density (B).** At high PDI (>0.2) immunity (A) and host population density (B) are uniformly high. Each point represents the median of the last 500 hours of a single simulation; all parameter sets from Table S1 are included. R^2^ correlation coefficients (A) 0.59 and (B) 0.78 are significant at p<0.001.
(EPS)

Figure S7 **HVI decreases with increasing PDI (A–C) and IDI (D–F).** PDI values binned by 0.1; IDI values binned by 0.6. Bars (and lines) are mean (and SEM) of median HVI across the last 500 hours of each replicate simulation from a pool of 100 simulations per parameter set. Parameters for each panel are (A,D) S = 10, P = 10, q = 10^−5^, μ = 5×10^−7^; (B,E) S = 10, P = 20, q = 10^−5^, μ = 5×10^−7^; (C,F) S = 10, P = 10, q = 10^−5^, μ = 10^−7^. All other parameters as listed in Table S2. R^2^ values (data not binned) are noted in each panel with *, p<0.01,;**, p<<0.001; NS, not significant.
(EPS)

Figure S8 **Example of methodology of CRISPR locus reconstruction from sequencing reads.** Each color represents a unique spacer. Each horizontal row on the left shows the spacer content of a single read; its corresponding row on the right shows the inferred complete spacer content. The spacer marked with an asterisk is not present in the ancestral host but has become fixed in the current population. L, leader sequence; T, spacers present in ancestral host.
(EPS)

Table S1 **Summary of simulated population outcomes.** Summary of the population outcomes (complete, viral extinction, unfilled locus) of simulations for each parameter set.
(DOCX)

Table S2 **Model parameters.** Description of parameters including symbol and value used for simulation of the model.
(DOCX)

Table S3 **Linear-quadratic model comparisons.** Summary of the R^2^ computation for Figure 4E–H and Figure S5E–H and choice of model fit using AIC.
(DOCX)

File S1 **Supplemental Information.** Includes a detailed description of the model used for simulation; a discussion of how host and viral strain size and immunity affect PDI; and a description of transient dynamics of hosts with limited immune history.
(DOC)

## References

[1] Breitbart M, Rohwer F (2005) Here a virus, there a virus, everywhere the same virus? Trends in Microbiology 13: 278–284 10.1016/j.tim.2005.04.003 15936660

[2] Suttle CA (2007) Marine viruses — major players in the global ecosystem. Nat Rev Micro 5: 801–812 10.1038/nrmicro1750 17853907

[3] Angly FE, Felts B, Breitbart M, Salamon P, Edwards RA, et al (2006) The marine viromes of four oceanic regions. PLoS Biol 4: e368 10.1371/journal.pbio.0040368 17090214 PMC1634881

[4] Labrie SJ, Samson JE, Moineau S (2010) Bacteriophage resistance mechanisms. Nature Reviews Microbiology 8: 317–327 10.1038/nrmicro2315 20348932

[5] Hyman P, Abedon ST (2010) Bacteriophage host range and bacterial resistance. Adv Appl Microbiol 70: 217–248 10.1016/S0065-2164(10)70007-1 20359459

[6] Bikard D, Marraffini LA (2012) Innate and adaptive immunity in bacteria: mechanisms of programmed genetic variation to fight bacteriophages. Current Opinion in Immunology 24: 15–20 10.1016/j.coi.2011.10.005 22079134

[7] Wilson GG, Murray NE (1991) Restriction and modification systems. Annual Review of Genetics 25: 585–627 10.1146/annurev.ge.25.120191.003101 1812816

[8] Tock MR, Dryden DT (2005) The biology of restriction and anti-restriction. Current Opinion in Microbiology 8: 466–472 10.1016/j.mib.2005.06.003 15979932

[9] Molineux IJ (1991) Host-parasite interactions: recent developments in the genetics of abortive phage infections. New Biol 3: 230–236. 1831658

[10] Chopin M-C, Chopin A, Bidnenko E (2005) Phage abortive infection in lactococci: variations on a theme. Current Opinion in Microbiology 8: 473–479 10.1016/j.mib.2005.06.006 15979388

[11] Ayala FJ, Campbell CA (1974) Frequency-dependent selection. Annual Review of Ecology and Systematics 5: 115–138 10.1146/annurev.es.05.110174.000555

[12] Levin BR, Antonovics J, Sharma H (1988) Frequency-dependent selection in bacterial populations [and discussion]. Phil Trans R Soc Lond B 319: 459–472 10.1098/rstb.1988.0059 2905487

[13] Buckling Rainey, Travisano Kassen (2000) The emergence and maintenance of diversity: insights from experimental bacterial populations. Trends Ecol Evol 15: 243–247. 10802550 10.1016/s0169-5347(00)01871-1

[14] Winter C, Bouvier T, Weinbauer MG, Thingstad TF (2010) Trade-offs between competition and defense specialists among unicellular planktonic organisms: the “killing the winner” hypothesis revisited. Microbiol Mol Biol Rev 74: 42–57 10.1128/MMBR.00034-09 20197498 PMC2832346

[15] Weitz JS, Hartman H, Levin SA (2005) Coevolutionary arms races between bacteria and bacteriophage. PNAS 102: 9535–9540 10.1073/pnas.0504062102 15976021 PMC1172273

[16] Lenski RE (1988) Experimental studies of pleiotropy and epistasis in *Escherichia coli*. I. Variation in competitive fitness among mutants resistant to virus T4. Evolution 42: 425–432 10.2307/2409028 28564005

[17] Bohannan BJM, Lenski RE (1997) Effect of resource enrichment on a chemostat community of bacteria and bacteriophage. Ecology 78: 2303–2315 10.2307/2265893

[18] Buckling A, Rainey PB (2002) Antagonistic coevolution between a bacterium and a bacteriophage. Proc Biol Sci 269: 931–936 10.1098/rspb.2001.1945 12028776 PMC1690980

[19] Buckling A, Wei Y, Massey RC, Brockhurst MA, Hochberg ME (2006) Antagonistic coevolution with parasites increases the cost of host deleterious mutations. Proc R Soc B 273: 45–49 10.1098/rspb.2005.3279 PMC1560003 16519233

[20] Lopez-Pascua L d C, Buckling A (2008) Increasing productivity accelerates host-parasite coevolution. J Evol Biol 21: 853–860 10.1111/j.1420-9101.2008.01501.x 18284514

[21] Forde SE, Thompson JN, Holt RD, Bohannan BJM (2008) Coevolution drives temporal changes in fitness and diversity across environments in a bacteria-bacteriophage interaction. Evolution 62: 1830–1839 10.1111/j.1558-5646.2008.00411.x 18452575

[22] Barrangou R, Fremaux C, Deveau H, Richards M, Boyaval P, et al (2007) CRISPR provides acquired resistance against viruses in prokaryotes. Science 315: 1709–1712 10.1126/science.1138140 17379808

[23] Barrangou R, Horvath P (2012) CRISPR: new horizons in phage resistance and strain identification. Annu Rev Food Sci Technol 3: 143–162 10.1146/annurev-food-022811-101134 22224556

[24] Deveau H, Garneau JE, Moineau S (2010) CRISPR/Cas system and its role in phage-bacteria interactions. Annu Rev Microbiol 64: 475–493 10.1146/annurev.micro.112408.134123 20528693

[25] Deveau H, Barrangou R, Garneau JE, Labonté J, Fremaux C, et al (2008) Phage response to CRISPR-encoded resistance in *Streptococcus thermophilus* . J Bacteriol 190: 1390–1400 10.1128/JB.01412-07 18065545 PMC2238228

[26] Horvath P, Romero DA, Coûté-Monvoisin A-C, Richards M, Deveau H, et al (2008) Diversity, activity, and evolution of CRISPR loci in *Streptococcus thermophilus* . J Bacteriol 190: 1401–1412 10.1128/JB.01415-07 18065539 PMC2238196

[27] Horvath P, Barrangou R (2010) CRISPR/Cas, the immune system of bacteria and archaea. Science 327: 167–170 10.1126/science.1179555 20056882

[28] Marraffini LA, Sontheimer EJ (2010) CRISPR interference: RNA-directed adaptive immunity in bacteria and archaea. Nat Rev Genet 11: 181–190 10.1038/nrg2749 20125085 PMC2928866

[29] Terns MP, Terns RM (2011) CRISPR-based adaptive immune systems. Curr Opin Microbiol 14: 321–327 10.1016/j.mib.2011.03.005 21531607 PMC3119747

[30] Van der Oost J, Jore MM, Westra ER, Lundgren M, Brouns SJJ (2009) CRISPR-based adaptive and heritable immunity in prokaryotes. Trends in Biochemical Sciences 34: 401–407 10.1016/j.tibs.2009.05.002 19646880

[31] Westra ER, Swarts DC, Staals RHJ, Jore MM, Brouns SJJ, et al (2012) The CRISPRs, they are a-changin': how prokaryotes generate adaptive immunity. Annu Rev Genet 46: 311–339 10.1146/annurev-genet-110711-155447 23145983

[32] Al-Attar S, Westra ER, van der Oost J, Brouns SJJ (2011) Clustered regularly interspaced short palindromic repeats (CRISPRs): the hallmark of an ingenious antiviral defense mechanism in prokaryotes. Biological Chemistry 392: 277–289. 21294681 10.1515/BC.2011.042

[33] Grissa I, Vergnaud G, Pourcel C (2007) CRISPRFinder: a web tool to identify clustered regularly interspaced short palindromic repeats. Nucleic Acids Research 35: W52–W57 10.1093/nar/gkm360 17537822 PMC1933234

[34] Mojica FJM, Díez-Villaseñor C, García-Martínez J, Soria E (2005) Intervening sequences of regularly spaced prokaryotic repeats derive from foreign genetic elements. J Mol Evol 60: 174–182 10.1007/s00239-004-0046-3 15791728

[35] Mojica FJM, Díez-Villaseñor C, García-Martínez J, Almendros C (2009) Short motif sequences determine the targets of the prokaryotic CRISPR defence system. Microbiology 155: 733–740 10.1099/mic.0.023960-0 19246744

[36] Brouns SJJ, Jore MM, Lundgren M, Westra ER, Slijkhuis RJH, et al (2008) Small CRISPR RNAs guide antiviral defense in prokaryotes. Science 321: 960–964 10.1126/science.1159689 18703739 PMC5898235

[37] Levin BR, Moineau S, Bushman M, Barrangou R (2013) The population and evolutionary dynamics of phage and bacteria with CRISPR-mediated immunity. PLoS Genet 9: e1003312 10.1371/journal.pgen.1003312 23516369 PMC3597502

[38] Sun CL, Barrangou R, Thomas BC, Horvath P, Fremaux C, et al (2013) Phage mutations in response to CRISPR diversification in a bacterial population. Environmental Microbiology 15: 463–470 10.1111/j.1462-2920.2012.02879.x 23057534

[39] Semenova E, Jore MM, Datsenko KA, Semenova A, Westra ER, et al (2011) Interference by clustered regularly interspaced short palindromic repeat (CRISPR) RNA is governed by a seed sequence. PNAS 108: 10098–10103 10.1073/pnas.1104144108 21646539 PMC3121866

[40] Desai MM, Fisher DS, Murray AW (2007) The speed of evolution and maintenance of variation in asexual populations. Current Biology 17: 385–394 10.1016/j.cub.2007.01.072 17331728 PMC2987722

[41] Gerrish PJ, Lenski RE (1998) The fate of competing beneficial mutations in an asexual population. Genetica 102–103: 127–144. 9720276

[42] Fisher RA (1930) The Genetical Theory Of Natural Selection. Oxford: Oxford University Press. 308 p.

[43] Lenski RE, Levin BR (1985) Constraints on the coevolution of bacteria and virulent phage: a model, some experiments, and predictions for natural communities. The American Naturalist 125: 585–602 10.2307/2461275

[44] Childs LM, Held NL, Young MJ, Whitaker RJ, Weitz JS (2012) Multiscale model of CRISPR-induced coevolutionary dynamics: diversification at the interface of Lamarck and Darwin. Evolution 66: 2015–2029 10.1111/j.1558-5646.2012.01595.x 22759281 PMC3437473

[45] He J, Deem MW (2010) Heterogeneous diversity of spacers within CRISPR (Clustered Regularly Interspaced Short Palindromic Repeats). Phys Rev Lett 105: 128102 10.1103/PhysRevLett.105.128102 20867676

[46] Levin BR (2010) Nasty viruses, costly plasmids, population dynamics, and the conditions for establishing and maintaining CRISPR-mediated adaptive immunity in bacteria. PLoS Genet 6: e1001171 10.1371/journal.pgen.1001171 21060859 PMC2965746

[47] Haerter JO, Sneppen K (2012) Spatial structure and Lamarckian adaptation explain extreme genetic diversity at CRISPR locus. mBio 3: e00126–12 10.1128/mBio.00126-12 22807565 PMC3413401

[48] Weinberger AD, Sun CL, Pluciński MM, Denef VJ, Thomas BC, et al (2012) Persisting viral sequences shape microbial CRISPR-based immunity. PLoS Comput Biol 8: e1002475 10.1371/journal.pcbi.1002475 22532794 PMC3330103

[49] Iranzo J, Lobkovsky AE, Wolf YI, Koonin EV (2013) Evolutionary dynamics of archaeal and bacterial adaptive immunity systems, CRISPR-Cas, in an explicit ecological context. J Bacteriol. doi:10.1128/JB.00412-13. 10.1128/JB.00412-13 PMC3754601 23794616

[50] Held NL, Childs LM, Davison M, Weitz JS, Whitaker RJ, et al.. (2013) CRISPR-Cas Systems to Probe Ecological Diversity and Host–Viral Interactions. In: Barrangou R, Oost J van der, editors. CRISPR-Cas Systems. Springer Berlin Heidelberg. pp. 221–250.

[51] Makarova KS, Aravind L, Wolf YI, Koonin EV (2011) Unification of Cas protein families and a simple scenario for the origin and evolution of CRISPR-Cas systems. Biol Direct 6: 38 10.1186/1745-6150-6-38 21756346 PMC3150331

[52] Paez-Espino D, Morovic W, Sun CL, Thomas BC, Ueda K, et al (2013) Strong bias in the bacterial CRISPR elements that confer immunity to phage. Nat Commun 4: 1430 10.1038/ncomms2440 23385575

[53] Touchon M, Rocha EPC (2010) The Small, Slow and Specialized CRISPR and Anti-CRISPR of *Escherichia* and *Salmonella* . PLoS ONE 5: e11126 10.1371/journal.pone.0011126 20559554 PMC2886076

[54] Rho M, Wu Y-W, Tang H, Doak TG, Ye Y (2012) Diverse CRISPRs evolving in human microbiomes. PLoS Genet 8: e1002441 10.1371/journal.pgen.1002441 22719260 PMC3374615

[55] Cui Y, Li Y, Gorgé O, Platonov ME, Yan Y, et al (2008) Insight into microevolution of *Yersinia pestis* by clustered regularly interspaced short palindromic repeats. PLoS ONE 3: e2652 10.1371/journal.pone.0002652 18612419 PMC2440536

[56] Held NL, Herrera A, Cadillo-Quiroz H, Whitaker RJ (2010) CRISPR associated diversity within a population of *Sulfolobus islandicus* . PLoS ONE 5: e12988 10.1371/journal.pone.0012988 20927396 PMC2946923

[57] Held NL, Herrera A, Whitaker RJ (2013) Reassortment of CRISPR repeat-spacer loci in *Sulfolobus islandicus*. Environ Microbiol. doi:10.1111/1462-2920.12146. 10.1111/1462-2920.12146 23701169

[58] Andersson AF, Banfield JF (2008) Virus population dynamics and acquired virus resistance in natural microbial communities. Science 320: 1047–1050 10.1126/science.1157358 18497291

[59] Tyson GW, Banfield JF (2008) Rapidly evolving CRISPRs implicated in acquired resistance of microorganisms to viruses. Environ Microbiol 10: 200–207 10.1111/j.1462-2920.2007.01444.x 17894817

[60] Drake JW (2009) Avoiding dangerous missense: thermophiles display especially low mutation rates. PLoS Genet 5: e1000520 10.1371/journal.pgen.1000520 19543367 PMC2688765

[61] Weinberger AD, Wolf YI, Lobkovsky AE, Gilmore MS, Koonin EV (2012) Viral diversity threshold for adaptive immunity in prokaryotes. MBio 3: e00456–12 10.1128/mBio.00456-12 23221803 PMC3517865

[62] Jiang W, Maniv I, Arain F, Wang Y, Levin BR, et al (2013) Dealing with the evolutionary downside of CRISPR immunity: bacteria and beneficial plasmids. PLoS Genet 9: e1003844 10.1371/journal.pgen.1003844 24086164 PMC3784566

[63] Gandon S, Vale PF (2014) The evolution of resistance against good and bad infections. J Evol Biol 27: 303–312 10.1111/jeb.12291 24329755

[64] Barrick JE, Yu DS, Yoon SH, Jeong H, Oh TK, et al (2009) Genome evolution and adaptation in a long-term experiment with *Escherichia coli* . Nature 461: 1243–1247 10.1038/nature08480 19838166

