# Supplementary material for: CRISPR-Induced Distributed Immunity in Microbial Populations
Source: PLoS One. 2014 Jul 7;9(7):e101710. doi: 10.1371/journal.pone.0101710 (PMC4084950; doi:10.1371/journal.pone.0101710)
Supplement: Table S1 — Summary of simulated population outcomes. Summary of the population outcomes (complete, viral extinction, unfilled locus) of simulations for each parameter set. (DOCX) [file pone.0101710.s009.docx]

**Table S1: Summary of simulated population outcomes.**

| **S** | **P** | **μ** | **q** | **Complete** | **Viral Extinction^*^** | **Unfilled Locus** | **Total** | **Simulation**  **Length (h)** |
| --- | --- | --- | --- | --- | --- | --- | --- | --- |
| 5 | 10 | 5.0E-07 | 1.0E-05 | 200 | 0 | 0 | 200 | 2500 |
| 10 | 5 | 5.0E-07 | 1.0E-05 | 200 | 0 | 0 | 200 | 2500 |
| 10 | 10 | 5.0E-07 | 1.0E-05 | 199 | 0 | 1 | 200 | 2500 |
| 10 | 15 | 5.0E-07 | 1.0E-05 | 183 | 2 | 15 | 200 | 10000 |
| 10 | 20 | 5.0E-07 | 1.0E-05 | 132 | 15 | 53 | 200 | 10000 |
| 10 | 10 | 1.0E-07 | 1.0E-05 | 29 | 4 | 167 | 200 | 2500 |
| 10 | 10 | 2.5E-07 | 1.0E-05 | 173 | 0 | 27 | 200 | 2500 |
| 10 | 10 | 7.5E-07 | 1.0E-05 | 200 | 0 | 0 | 200 | 2500 |
| 10 | 10 | 1.0E-06 | 1.0E-05 | 199 | 0 | 1 | 200 | 2500 |
| 10 | 10 | 5.0E-07 | 1.0E-06 | 148 | 16 | 36 | 200 | 2500 |
| 10 | 10 | 5.0E-07 | 5.0E-06 | 198 | 0 | 2 | 200 | 2500 |
| 10 | 10 | 5.0E-07 | 5.0E-05 | 200 | 0 | 0 | 200 | 2500 |
| 10 | 10 | 5.0E-07 | 1.0E-04 | 200 | 0 | 0 | 200 | 2500 |

* viral population falling below density cutoff (0.1/mL) during the last 500 hours of the simulation.
