# Supplementary material for: CRISPR-Induced Distributed Immunity in Microbial Populations
Source: PLoS One. 2014 Jul 7;9(7):e101710. doi: 10.1371/journal.pone.0101710 (PMC4084950; doi:10.1371/journal.pone.0101710)
Supplement: Table S2 — Model parameters. Description of parameters including symbol and value used for simulation of the model. (DOCX) [file pone.0101710.s010.docx]

# Table S2: Model parameters

| **Parameter** | **Description** | **Standard value** | **Other values** |
| --- | --- | --- | --- |
| p | CRISPR failure probability | 1.0e-05 | -- |
| q | spacer acquisition probability | 1.0e-05 | 1.0e-06, 5.0e-06, 5.0e-05, 1.0e-04 |
| r | growth rate (1/h) | 1 | -- |
| K | carrying capacity (1/mL) | 1.0e5.5 | -- |
| β | burst size | 50 | -- |
| Φ | adsorption rate (mL/h) | 1.0e-07 | -- |
| m | viral decay rate (1/h) | 0.1 | -- |
| μ | mutation rate | 5.0e-07 | 1.0e-07, 2.5e-07, 7.5e-07, 1.0e-06 |
| ρ | density cutoff (1/mL) | 0.1 | -- |
| S | number of spacers | 10 | 5 |
| P | number of protospacers | 10 | 5, 15, 20 |
