# Supplementary material for: CRISPR-Induced Distributed Immunity in Microbial Populations
Source: PLoS One. 2014 Jul 7;9(7):e101710. doi: 10.1371/journal.pone.0101710 (PMC4084950; doi:10.1371/journal.pone.0101710)
Supplement: Table S3 — Linear-quadratic model comparisons. Summary of the R2 computation for Figure 4E–H and Figure S5E–H and choice of model fit using AIC. (DOCX) [file pone.0101710.s011.docx]

**Table S3: Linear - quadratic model comparisons.**

| **Parameter** | **Type** | **Description** | **R^2^** | **AIC** | **p** | **Figure** |
| --- | --- | --- | --- | --- | --- | --- |
| **μ** | linear | PDI vs. viral population density | 0.10 | 26460 | <0.001 | 4E |
|  | quadratic | PDI vs. viral population density | 0.66 | 25674 | <0.001 | 4E |
| **P** | linear | PDI vs. viral population density | 0.01 | 23960 | <0.001 | 4F |
|  | quadratic | PDI vs. viral population density | 0.69 | 23129 | <0.001 | 4F |
| **μ** | linear | PDI vs. viral strain count | 0.19 | 10425 | <0.001 | 4G |
|  | quadratic | PDI vs. viral strain count | 0.20 | 10422 | 0.02 | 4G |
| **P** | linear | PDI vs. viral strain count | 0.06 | 8966 | <0.001 | 4H |
|  | quadratic | PDI vs. viral strain count | 0.40 | 8648 | <0.001 | 4H |
| **q** | linear | PDI vs. viral population density | 0.60 | 30410 | <0.001 | S5E |
|  | quadratic | PDI vs. viral population density | 0.67 | 30229 | <0.001 | S5E |
| **S** | linear | PDI vs. viral population density | 0.48 | 11988 | <0.001 | S5F |
|  | quadratic | PDI vs. viral population density | 0.57 | 11913 | <0.001 | S5F |
| **q** | linear | PDI vs. viral strain count | 0.20 | 11382 | <0.001 | S5G |
|  | quadratic | PDI vs. viral strain count | 0.26 | 11313 | <0.001 | S5G |
| **S** | linear | PDI vs. viral strain count | 0.00 | 4575 | 0.80 | S5H |
|  | quadratic | PDI vs. viral strain count | 0.00 | 4575 | 0.17 | S5H |
