# Supplementary material for: CRISPR-Induced Distributed Immunity in Microbial Populations
Source: PLoS One. 2014 Jul 7;9(7):e101710. doi: 10.1371/journal.pone.0101710 (PMC4084950; doi:10.1371/journal.pone.0101710)
Supplement: File S1 — Supplemental Information. Includes a detailed description of the model used for simulation; a discussion of how host and viral strain size and immunity affect PDI; and a description of transient dynamics of hosts with limited immune history. (DOC) [file pone.0101710.s012.doc]

# Supplemental Info for “CRISPR-induced distributed immunity in microbial populations”

**Authors: Lauren M. Childs,^*,1^ Whitney England,^*,2^ Mark J. Young,^3^ Joshua S. Weitz,^4^ Rachel J. Whitaker^5^**

***^*^ denotes equal contribution***

***^1^ Center for Communicable Disease Dynamics, Department of Epidemiology, Harvard School of Public Health, 677 Huntington Avenue, Boston, Massachusetts 02115***

***^2^ Department of Microbiology, University of Illinois at Urbana-Champaign, 601 S. Goodwin Avenue, Urbana, Illinois 61801***

***^3^ Thermal Biology Institute and Department of Plant Sciences and Plant Pathology, Montana State University, 307 Plant Bioscience Building, Bozeman, Montana 59717***

***^4^ School of Biology and School of Physics, Georgia Institute of Technology, 310 Ferst Drive, Atlanta, Georgia, 30332***

***^5^ Department of Microbiology and Institute for Genomic Biology, University of Illinois at Urbana-Champaign, 601 S. Goodwin Avenue, Urbana, Illinois 61801***

**Model Information**

The code for simulating eco-evolutionary dynamics has been modified from that described in (42) to optimize computational performance. A description of changes and the modified code are available on the Dryad Data Repository (http://doi.org/10.5061/dryad.2vm20*)*. Simulations were completed in Matlab (2009a/2011b/2012a MathWorks) using *ode45* with parameters found in Table S2.

Within this model, the intrinsic growth rate of all strains is identical while the net growth rate (births minus deaths) depends on the immunity relationships between hosts and viruses. Viruses can only replicate when there exists a susceptible host. Hosts can acquire new spacers during any interaction with a virus. When acquiring a spacer, if the host is already immune to the virus, it acquires another spacer matching the virus, but immunity remains unchanged. If the host is susceptible to the virus, it survives the interaction and is immune to subsequent infections with any virus that has the acquired spacer. Mathematical analysis of simple cases of the model are presented in Child*s et a*l. (2012).

This model of the CRISPR system includes a number of assumptions, which we discuss briefly here. Extensive discussion of model choices can be found in Childs *et al* (2012). Ecological parameters are chosen such that viruses will die out when infecting immune hosts and will coexist when infecting non-immune hosts. Immunity of a host is determined by the presence of at least one spacer matching a virus and does not increase with the acquisition of additional matching spacers. It is assumed that a single spacer is sufficient for complete immunity to viruses containing the matching protospacer. In order to facilitate computation, we assume that spacer loci are of a pre-defined fixed length with acquisition of a new (leader) end spacer balanced by removal of an old (trailer) end spacer when a locus is full. Further, we focus on parameters such that the number of protospacer is always at or greater than the maximal number of spacers to preserve a high relative number of spacers to protospacers as observed in natural systems. Our standard values for the viral mutation rate and host spacer acquisition rate are estimated from CRISPR-mediated host-viral interactions in a *Streptococcus thermophilus* system (Barrangou et al., 2007).

**Host and viral strain size and immunity affect PDI**

The computation of PDI is affected by the number and size of host and viral strains as well as the immunity relationships between these strains. Although all triplets of two host strains and a viral strain are considered in the computation, triplets where identical host strains are represented twice will not contribute to PDI.

*Effect of host strain size and number*

The simplest population structure where non-zero PDI is possible occurs with two host strains and a single viral strain (Figure 1). Assuming both host strains are immune to the viral strain in unique ways, the level of PDI depends on the abundances of the host strains. When the two host strains are present in equal proportions (N_1_=0.5, N_2_=0.5), then PDI is 0.5, the maximum possible with 2 strains (Figure S1). However, when one strain is dominant then the PDI is much below the maximum. For example, if one host is 90% of the host population (N_1_=0.9 and N_2_=0.1), PDI is only 0.18 (Figure S1).

When there are three hosts, which are all immune to the single viral strain in unique ways, the maximum PDI increases to 0.66 (Figure S1), when all three strains have equal abundances (N_1_=N_2_=N_3_=0.33). When one strain dominates (for example, N_1_=.9, N_2_=.1, N_3_=.1), the PDI is much below the maximum (Figure S1). As the number of host strains increases, the maximum PDI value continues to increase toward the absolute maximum value of 1 but the actual PDI value depends on the distribution of host strain sizes (Figure S1).

*Effect of viral strain size and number*

Unlike in the case of hosts, the number of viral strains does not directly affect the value of PDI. However, as the viral population becomes more fragmented, there are more immunity interactions to consider. For example in a population with two host strains and two viral strains, if both hosts are immune to the two viral strains in different ways, then the level of PDI is dependent solely on population abundances of N_1_ and N_2_ as in the single viral strain scenario. However, if the two host strains are only immune to one virus in unique ways or not immune at all than the level of PDI is only a fraction of the single viral case.

As the viral population is further fragmented, each set of relationships between two hosts and each virus needs to be evaluated separately for unique spacer matches. Although each comparison potentially contributes to PDI, as there are more viruses, the individual contribution of each comparison is small. Further, the total portion of the viral population matched by the host population influences the maximum PDI value, since there cannot be high PDI when immunity is low.

**Transient dynamics of hosts with limited immune history**

We observe transient dynamics during the initial period of our simulation while the spacer locus goes from zero spacers per host on average to the maximum spacers per host, *S*. The length of this transient dynamic depends on the parameters but always occurs well below the time period we use to compute our output statistics. With low protospacer numbers, the transient period is typically under 500 hours but increases to thousands of hours as the protospacer number is increased. This period is a result of seeding all our simulations with a single host strain and a single viral strain where the host strain is susceptible to viral infection.

Initially the viral population is capable of proliferating during all interactions with the host population and rapidly does so (see Figure S2). During this proliferation several neutral viral mutants, each involving single protospacer changes, emerge at low density and grow at an identical rate to the original viral strain. As only susceptible hosts exist there is no immediate advantage to the mutations.

On a similar time scale the susceptible host population begins to decline due to viral proliferation; the larger viral population results in an increased number of host-viral interactions. However, due to the large number of host-viral interactions, host mutation (spacer acquisition) events occur, where new host strains emerge, each acquiring immunity to nearly all viruses. These host mutants are introduced into the population at low density and grow to large density with their significantly enhanced survival rate compared to the original host strain, which is killed during every viral interaction (see Figure S2). For example in Figure S2, a single host strain is created from the original susceptible strain at 3 hours that is immune to most of the viral population, has an enhanced net growth rate and nearly sweeps the population by 20 hours. Due to their fitness advantages, the mutant hosts take over the population with a total abundance reaching nearly the host carrying capacity *K*. As the mutant hosts increase toward carrying capacity, the viral population decreases given the rapidly decreasing number of susceptible hosts on which viruses can proliferate.

The rapid switch from the original host to the mutant hosts at a population level results in shift in the average number of relevant spacers per host from zero to one (Figure S2c). Through this rapid host transition, the virus population continued to decrease. If the virus population has not already created mutants and is unable to produce a mutant, the viral population dies out (not pictured but quantified in Table S1). Otherwise extant mutants begin to grow or new mutants are produced and begin to grow (Figure S2). As the host-viral interactions again increase, the host population acquires additional host mutants with two spacers per host. Hosts with multiple immune matches to viruses and their progeny increase in the host population (Figure S2). Despite this, some viruses remain able to grow on a subset of the host population, and thus produce new mutants. Selection for particular host and viral strains due to the immunity relationships occurs, and hosts with a single spacer become obsolete leading to an increase in the average number of spacers per host from one to two (Figure S2c).

As more host and viral mutants are created, the immunity relationships become more complicated and the selection for host and viral strains by the relationships and population densities of existing strains loses a defined structure. The growth of any particular strain is dependent upon its immunity relationships and its population density compared to other strains in the population. We take population level averages after the spacer locus of all hosts has been filled for at least several hundred hours

**Supplemental References**

Barrangou R, Fremaux C, Deveau H, Richards M, Boyaval P, Moineau S, et al. (2007). CRISPR provides acquired resistance against viruses in prokaryotes. Science 315:1709–1712.

Childs LM, Held NL, Young MJ, Whitaker RJ, Weitz JS. (2012). Multiscale model of CRISPR-induced coevolutionary dynamics: diversification at the interface of Lamarck and Darwin. Evolution 66:2015–2029.
